# Surgery Alone Treatment vs. Surgery with Adjuvant Therapy for Laryngeal Mucoepidermoid Cancer: A Systematic Review

**DOI:** 10.32604/or.2026.073086

**Published:** 2026-03-23

**Authors:** Francesco Chiari, Giovanni Motta, Daria Maria Filippini, Claudio Donadio Caporale, Pierre Guarino

**Affiliations:** 1 Otolaryngology Head and Neck Unit, “Santo Spirito” Hospital, Pescara, Italy; 2 Head and Neck Surgery Unit, University of Campania “Luigi Vanvitelli”, Naples, Italy; 3 Medical Oncology Unit, IRCCS Azienda Ospedaliero Universitaria of Bologna, Bologna, Italy

**Keywords:** Laryngeal carcinoma, mucoepidermoid cancer (MEC), head and neck, salivary-type carcinoma, adjuvant radiotherapy, surgery, oncologic outcomes, rare head and neck tumors

## Abstract

**Objective:**

Mucoepidermoid carcinoma (MEC) of the larynx is an extremely rare malignancy, accounting for less than 1% of primary laryngeal tumors. The optimal role of adjuvant therapy, particularly radiotherapy (RT), remains unclear due to limited evidence. This systematic review aimed to evaluate oncologic outcomes and the impact of adjuvant treatment in patients with early- and advanced-stage laryngeal MEC.

**Methods:**

A systematic literature search was performed according to PRISMA 2020 guidelines in PubMed/Embase, Scopus, and Cochrane for studies published up to 31 July 2025.

**Results:**

Twenty-two studies, encompassing 55 patients, were included. Early-stage (T1–T2) patients (n = 28) treated with surgery alone achieved a 5-year local control of disease (LCD) of 85%–88% and disease-free survival (DFS) of 77%, whereas those receiving adjuvant RT showed 100% LCD and DFS, although differences were not statistically significant. In advanced-stage (T3–T4) patients (n = 27), adjuvant RT was associated with improved 2- and 5-year LCD reached 100% vs. 56% and 38% in surgery-only patients (OR 0.59; 95% CI, 0.34–0.83; *p* = 0.012). DFS at 5 years was 80% in the adjuvant group compared with 36% in surgery alone.

**Conclusions:**

Surgical excision with negative margins remains the cornerstone of treatment for early-stage laryngeal MEC, with limited added benefit from adjuvant RT. In advanced-stage or high-grade disease, postoperative RT significantly improves LCD and may enhance DFS. Chemotherapy remains reserved for rare, high-risk cases.

## 1 Introduction

Mucoepidermoid carcinoma (MEC) is the most common malignant tumor of the salivary glands, accounting for approximately 5%–10% of all salivary gland malignancies [1,2]. Its occurrence in the larynx, however, is exceptionally rare, representing less than 1% of all primary malignant laryngeal neoplasms [2–4]. Chiari et al. [5] reported the most recent systematic review evaluating the clinical characteristics and prognostic outcomes of laryngeal MEC.

The mean age at presentation is between the fifth and seventh decades of life, although pediatric and adolescent cases have also been described [6,7]. There appears to be no clear gender predilection, although some reports suggest a slight male predominance [3,4,8]. The supraglottis is the most frequently affected subsite, followed by the glottis and subglottis [3,8–10]. Patients typically present with nonspecific symptoms such as hoarseness, dysphagia, dyspnea, or airway obstruction, which often mimic those of squamous cell carcinoma (SCC) of the larynx [10–12].

Histopathologically, laryngeal MEC demonstrates a wide spectrum of biological behavior. Low-grade tumors are often well circumscribed and associated with favorable outcomes, whereas high-grade lesions exhibit aggressive features including infiltrative growth, necrosis, and high mitotic activity [3,8,13]. Histologic grade has consistently been reported as the strongest prognostic factor: 5-year disease survival exceeds 80%–90% for low-grade MEC, whereas high-grade tumors are associated with significantly poorer outcomes, with survival rates often below 50% [3,4,8]. Additional adverse prognostic factors include positive or close surgical margins, perineural invasion, lymphovascular invasion, cervical lymph node metastasis, and extracapsular spread [1,2,4].

Surgical resection remains the primary treatment modality, similar to the management of laryngeal SCC. Depending on tumor size and location, procedures range from endoscopic cordectomy and partial laryngectomy to total laryngectomy [3,9,12,13]. To date, the role of minimally invasive surgical approaches, such as transoral laser microsurgery (TOLM) or transoral robotic surgery (TORS), has not been systematically described for laryngeal MEC [14–16]. Moreover, the role of adjuvant therapy, particularly postoperative radiotherapy (RT) with or without chemotherapy (CHT), remains less well established. Although RT is frequently administered in patients with high-grade tumors, positive margins, or nodal disease, evidence supporting a definitive survival benefit remains limited to retrospective studies and anecdotal reports [1,4,13]. To date, only six cases of laryngeal MEC managed exclusively with definitive RT have been reported in the literature. All involved supraglottic or glottic early-stage MEC and achieved complete response, suggesting that RT may represent a feasible option in highly selected cases [1,9,17–19].

Given the rarity of this malignancy, the current literature is fragmented and lacks prospective, controlled studies. Most available data derive from case reports or small retrospective cohorts, precluding strong evidence-based recommendations. In this context, a systematic review is warranted to critically evaluate the impact of adjuvant therapy on survival and disease control in patients with laryngeal MEC.

Moreover, the rationale for adjuvant therapy in laryngeal MEC, particularly postoperative RT with or without CHT, remains poorly defined and supported only by heterogeneous retrospective evidence, underlining the urgent need for clarification. In this context, the aim of this systematic review is to assess the role of adjuvant therapy in the management of MEC, with particular attention to survival outcomes, local control, and recurrence rates.

## 2 Material and Methods

### 2.1 Search Strategy and Information Sources

The systematic literature search followed PRISMA 2020 guidelines [20]. A PRISMA 2020 flow diagram is provided in Supplementary Materials Tables S1 and S2. Searches were conducted in PubMed/Embase, Scopus, and Cochrane Library for articles published up to 31 July 2025. The final search strategy included both free-text terms and controlled vocabulary to capture historical and synonymous nomenclature for mucoepidermoid carcinoma of the larynx: (“mucoepidermoid carcinoma” OR “mucoepidermoid tumor” OR “mucoepidermoid neoplasm” OR “salivary gland-type carcinoma” OR “salivary-type carcinoma” OR “sialogenic carcinoma” OR “salivary gland malignancy”) AND (“larynx” OR “laryngeal” OR “glottis” OR “supraglottic” OR “subglottic”). Controlled vocabulary terms were also applied where available (e.g., [Mucoepidermoid Carcinoma] and [Laryngeal Neoplasms] in PubMed MeSH; ‘mucoepidermoid carcinoma’/exp and ‘larynx tumor’/exp in Embase). Duplicate records were automatically removed using EndNote v21 (Clarivate Analytics, Philadelphia, PA, USA) and verified manually. Only English-language and human studies were included, with no restrictions on study design. The complete, database-specific search strategies and retrieval numbers are provided in Supplementary Materials Table S3.

### 2.2 Study Selection and Data Extraction

The abstracts and titles obtained were screened independently by two of the authors (F. C., and P. G.), who afterwards met and discussed disagreements on citation inclusion. Discrepancies regarding citation inclusion were resolved through discussion and consensus. The eligibility of studies was assessed based on the PICOTS criteria [21], which included:

– Participants (patients affected by laryngeal MEC);
– Intervention (patients treated with surgery alone and surgery with adjuvant therapy);
– Comparator (comparison between patients treated with surgery alone and those treated with surgery + adjuvant therapy);
– Outcomes (oncologic outcomes, such as local control of disease (LCD), regional control of disease (RCD), distant control of disease (DCD), disease free survival (DFS), disease specific survival (DSS), and overall survival (OS));
– Time (1971–2025);
– Study design (systematic review).

**Figure 1:**
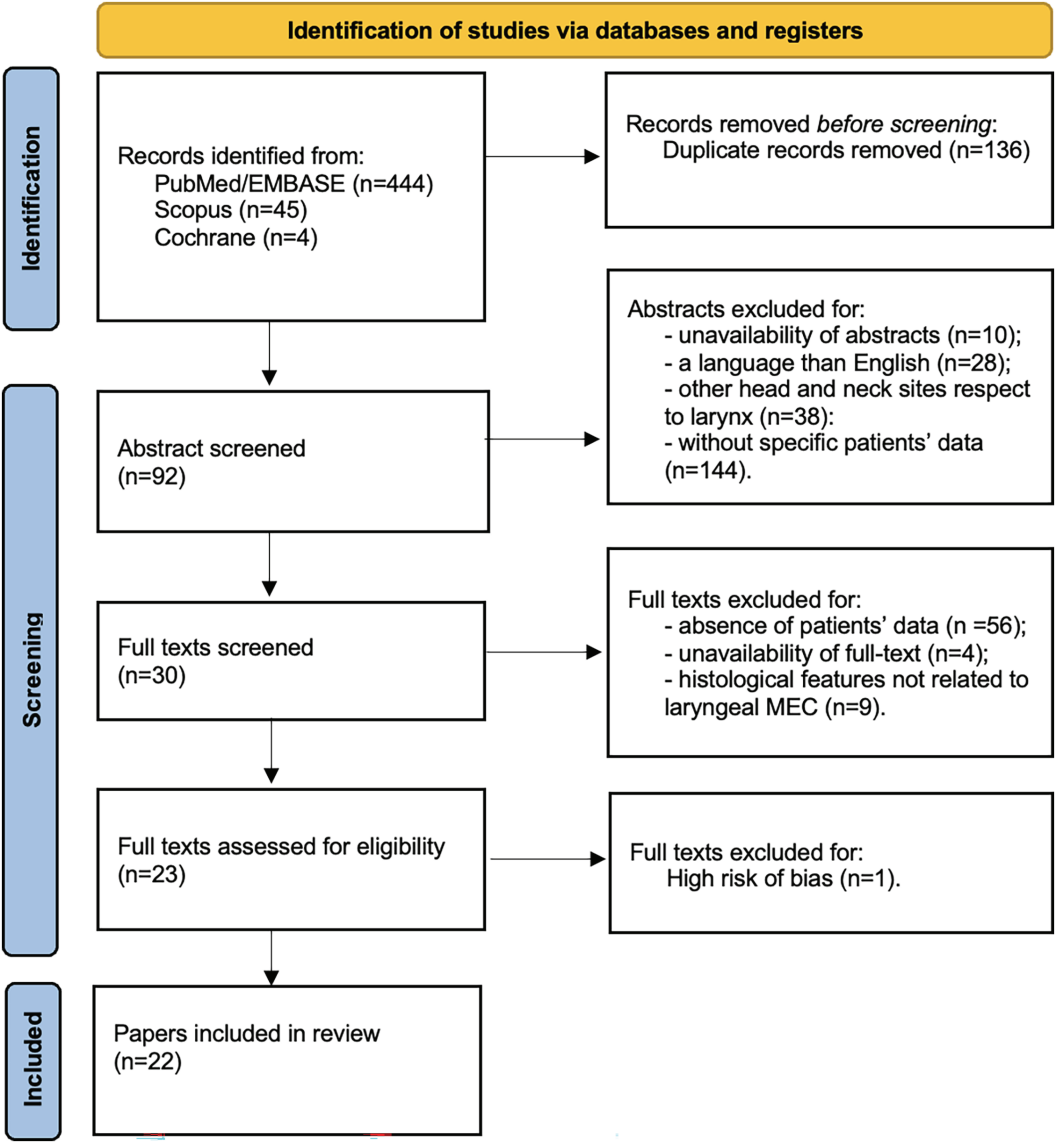
PRISMA 2020 flow chart of the study.

Inclusion criteria for abstract selection were the adoption of the English language, an abstract availability, oncologic outcomes (LCD, RCD, DCD, DFS, DSS, and OS) of patients affected by laryngeal MEC treated with surgical procedures with or without adjuvant therapies. The same two authors screened the full texts identified by such criteria, and then they met again and discussed disagreements on article inclusion. Inclusion criteria for full-text selected articles were the same as described above for abstract selection. Exclusion criteria included non-English articles, studies without abstract or full text, non-original works (reviews, letters, conference abstracts), absence of oncologic outcomes, tumors other than laryngeal MEC, and studies not involving surgical treatment with or without adjuvant therapy. A further manual check of the references included in the articles was performed. Details on the study selection process are reported in the PRISMA flow chart (Fig. 1). To avoid overlap with a previous review performed by Chiari et al. [5], particular attention was paid to the inclusion/exclusion process, ensuring that the current dataset reflects only studies meeting the revised eligibility criteria and updated search period. The current review was registered on PROSPERO database, nr: CRD420251238942.

Given the historical nature of several included studies, particular attention was paid to potential case overlap, as older reports sometimes incorporated cases previously published in earlier literature. To avoid duplication, a structured verification process was implemented. All studies were carefully cross-checked for overlapping patient characteristics, including institution and country of origin, year or period of treatment, patient demographics (age and sex), tumor subsite, stage, histological grade, and treatment modality. Articles identified as secondary literature or lacking original cases were excluded. In scenarios where overlap could not be fully excluded, the most detailed and recent description was retained and earlier reports were not used for study-level aggregated data extraction. After applying this cross-verification protocol, no duplicated cases were identified among the final cohort of patients.

### 2.3 Quality Assessment

Two authors (F. C. and P. G.) independently assessed the quality of the included studies using the methodological tool proposed by Murad et al. (BMJ Evidence-Based Medicine, 2018) [22], which is specifically designed for case reports and case series. This instrument evaluates four major domains—selection, ascertainment, causality, and reporting—through eight structured questions, allowing a practical and reproducible grading of bias risk (low, moderate, or high). Given that the majority of studies included in this review were case reports or small series, this tool was considered the most appropriate for assessing their internal validity. To strengthen methodological consistency across heterogeneous study designs, the NIH Quality Assessment Tool for Case Series Studies (2014) was additionally applied as a complementary framework [23] was also applied as a cross-validation framework, confirming the overall methodological adequacy and reliability of the included evidence. Studies with a high risk of bias according to Murad’s criteria were excluded from the analysis. The final distribution of risk-of-bias ratings for the included articles is presented in Supplementary Materials Table S4. The overall certainty of evidence was qualitatively evaluated using the GRADE framework (Grading of Recommendations, Assessment, Development and Evaluation), taking into account the domains of risk of bias, inconsistency, indirectness, imprecision, and publication bias. Given that most included studies were case reports or small case series, the certainty of evidence was rated as very low, mainly due to small-study effects and potential case-report bias. These study designs inherently provide limited generalizability and are prone to selective reporting, and are therefore consistently classified as very low–certainty evidence in established grading frameworks such as GRADE.

### 2.4 Data Analysis

Study-level aggregated data were extracted and summarized for descriptive and inferential analysis. Continuous variables were expressed as mean ± standard deviation (SD) or median (interquartile range, IQR), according to their distribution, and categorical variables as frequencies and percentages. The Shapiro–Wilk test was used to assess normality. Comparisons between categorical variables were performed using Fisher’s exact test or the *χ*^2^ test, as appropriate. Given the rarity of the disease and the limited sample size, all survival and disease-control outcomes were analyzed as time-to-event endpoints. Kaplan–Meier curves were generated for each outcome, and differences between groups were assessed using two-sided log-rank tests (α = 0.05). KM-derived survival and disease-control probabilities at fixed time points (2 and 5 years) were reported with their corresponding 95% confidence intervals (CIs), calculated using Greenwood’s variance and log–log transformation to constrain bounds within the 0%–100% range. When feasible, Firth-penalized Cox proportional hazards models were considered to account for sparse events and small subgroup sizes. In instances where individual time-to-event data were incomplete or insufficient for Cox modeling, fixed-time risk ratios and absolute risk differences at 2 and 5 years were calculated as descriptive sensitivity measures, applying the Haldane–Anscombe correction and exact tests when appropriate. Crude, non–time-adjusted proportions of recurrence and mortality were also summarized for descriptive purposes only, as they are not directly comparable with the KM-based estimates due to censoring. All statistical analyses were performed using STATA version 14 (StataCorp LLC, College Station, TX, USA). Tumor stage was classified according to the pathologic (pT/pN) criteria of the AJCC Cancer Staging Manual, 8th edition; when both clinical and pathologic stages were available, the pathologic classification was used for consistency. Because most included studies were case reports or small case series, individual-level survival times were generally unavailable. Kaplan–Meier curves in this review were therefore reconstructed from aggregated follow-up information and event counts. As such, KM estimates are intended solely as descriptive summaries of available data and should not be interpreted as inferential survival analysis. All time-to-event metrics derived from these curves must be considered exploratory and hypothesis-generating.

## 3 Results

A total of 494 manuscripts were identified across the databases, including 444 from PubMed/Embase, 45 from Scopus, and 5 from the Cochrane Library. After removal of duplicates, 358 unique records were retained. Following abstract screening, 92 articles were selected for full-text review, while 220 were excluded due to absence of abstract (n = 10), non-English language (n = 28), tumor site other than the larynx (n = 38), or absence of specific patients’ data (n = 144). Full-text screening further restricted eligibility to 30 articles. Of these, 56 were excluded due to lack of specific patients’ data, 4 due to unavailability of full text, and 9 due to histology not consistent with laryngeal MEC. Following risk-of-bias assessment, one additional article was excluded. Ultimately, 22 studies were included in the analysis (Fig. 1). These were published between 1975 and 2023 and collectively reported on 55 patients (Table 1). Database-specific strategies, hit counts, and deduplication details are provided in Supplementary Materials Table S2.

**Table 1:** Baseline characteristics of included patients (n = 55).

		Total Patients (55 pts)	Early Stage (28 pts)	Advanced Stage (27 pts)	
Category	Variable	Nr (Range/%)	Nr (Range/%)	Nr (Range/%)	*p-*Value
Patient features	Mean age in years, (±SD)	57.04 (±15.07)	56.34 (±12.37)	57.15 (±14.47)	0.55
	Males, n (%)	42 (76%)	23 (82%)	19 (70%)	0.36
	Females, n (%)	13 (24%)	5 (18%)	8 (30%)	0.36
Risk factors	Smoke, n/N (%)	6/9 (67%)	4/5 (80%)	2/4 (50%)	0.34
	Alcohol, n/N (%)	8/9 (89%)	5/5 (100%)	3/4 (75%)	0.24
Symptoms and signs	Hoarseness, n/N (%)	18/23 (78%)	10/12 (83%)	8/11 (73%)	0.54
	Dyspnoea, n/N (%)	4/23 (17%)	3/12 (25%)	1/11 (9%)	0.38
	Painful swallowing, n/N (%)	3/23 (13%)	1/12 (8%)	2/11 (18%)	0.48
	Neck mass, n/N (%)	3/23 (13%)	0/12 (0%)	3/11 (27%)	0.05
	Sore throat, n/N (%)	1/23 (4%)	1/12 (8%)	0/11 (0%)	0.33
	Haemoptysis, n/N (%)	1/23 (4%)	0/12 (0%)	1/11 (9%)	0.29
	Stridor, n/N (%)	1/23 (4%)	0/12 (0%)	1/11 (9%)	0.29
Laryngeal sub-sites	Supraglottic, n (%)	38 (69%)	15 (54%)	23 (85%)	0.02
	Glottic, n (%)	12 (22%)	9 (32%)	3 (11%)	0.05
	Subglottic, n (%)	5 (9%)	4 (14%)	1 (4%)	0.17
Surgical approach	Total laryngectomy, n (%)	33 (60%)	13 (46%)	20 (74%)	0.32
	Partial laryngectomy, n (%)	16 (29%)	11 (39%)	5 (19%)	0.32
	Cordectomy, n (%)	4 (7%)	3 (11%)	1 (4%)	0.32
	Epiglottectomy, n (%)	2 (4%)	1 (4%)	1 (4%)	0.32
	Cervical node dissection, n (%)	25 (45%)	5 (18%)	20 (74%)	<0.001
	Early T stage (T1-T2), n (%)	32 (58%)	28 (100%)	4 (15%)	<0.001
Pathological stage of disease	Advanced T stage (T3-T4), n (%)	23 (42%)	0 (0%)	23 (85%)	<0.001
	Lymph node metastasis (N+), n (%)	14 (25%)	0 (0%)	15 (56%)	<0.001
	G3 tumors, n (%)	12/31 (39%)	6/17 (36%)	6/14 (43%)	0.04
Type of adjuvant therapies	Adjuvant therapies, n (%)	15 (27%)	4 (14%)	11 (40%)	0.21
	- Adjuvant CHT, n (%)	4 (7%)	2 (7%)	2 (7%)	0.21
	- Adjuvant RT, n (%)	11 (20%)	3 (11%)	8 (30%)	0.21

Note: Abbreviations: CHT = chemotherapy; N = lymph-node metastasis; M = distant metastasis; nr = number; pts = patients; RT = radiotherapy; T = primary tumor. Note: Variables with incomplete reporting, such as risk factors and presenting symptoms, are presented as n/N (%), where N indicates the number of evaluable patients. Percentages for all other variables are based on the total cohort (N = 55).

### 3.1 Population Characteristics

A total of 55 patients across 22 studies were systematically reviewed (Table 1) [1–4,6–13,18,19,24–32]. The largest cohort included 11 patients [3]. Key demographic and clinical characteristics are summarized in Table 2. Of the included patients, 42 (76%) were males and 13 (24%) females, with a mean age of 57.04 years (SD ±15.7; range, 13–77 years). Regarding risk factors, information was available for 9 patients, of whom 6/9 (67%) reported smoking and 8/9 (89%) reported alcohol consumption. The most frequent presenting symptom was hoarseness, reported in 18 patients (78%), followed by dyspnoea (n = 4, 17%), odynophagia (n = 3, 13%), and cervical mass (n = 3, 13%). Less common symptoms included sore throat (n = 1, 4%), hemoptysis (n = 1, 4%), and stridor (n = 1, 4%). Tumor localization was most common in the supraglottis (n = 38/55, 69%), followed by the glottis (n = 12/55, 22%) and subglottis (n = 5/55, 9%). Surgical treatment consisted of total laryngectomy in 33 patients (60%), partial laryngectomy in 16 (29%), cordectomy in 4 (7%), and epiglottectomy in 2 (4%). Cervical lymph node dissection was performed in 25 cases (45%). Pathologic staging revealed early T-stage disease (T1–T2) in 32 patients (58%) and advanced T-stage disease (T3–T4) in 23 (42%), according to the AJCC Cancer Staging Manual, 8th edition [33]. Nodal metastasis was identified in 14 patients (25%). High-grade tumors (G3) were reported in 12 cases (39%). Adjuvant therapy was administered in 15 patients (27%), of whom 11 (20%) received RT and 4 (7%) CHT.

**Table 2:** Kaplan–Meier estimates of disease control and survival outcomes at 2 and 5 years according to stage and treatment modality.

Endopoint	Subgroup	2-Year KM Estimate (95% CI)	5-Year KM Estimate (95% CI)	Log-Rank p Value
LCD	Early-stage − Surgery alone	92 (84–100)	85 (75–95)	0.31
	Early-stage + Adjuvant therapy	100 (95–100)	100 (95–100)	
	Advanced-stage − Surgery alone	56 (40–72)	38 (22–54)	0.01
	Advanced-stage + Adjuvant therapy	100 (95–100)	100 (95–100)	
RCD	Early-stage − Surgery alone	88 (76–100)	82 (68–96)	0.28
	Early-stage + Adjuvant therapy	100 (95–100)	100 (95–100)	
	Advanced-stage − Surgery alone	72 (55–89)	56 (36–76)	0.09
	Advanced-stage + Adjuvant therapy	100 (95–100)	100 (95–100)	
DCD	Early-stage − Surgery alone	92 (82–100)	88 (76–100)	0.46
	Early-stage + Adjuvant therapy	100 (95–100)	100 (95–100)	
	Advanced-stage − Surgery alone	82 (68–96)	78 (62–94)	0.33
	Advanced-stage + Adjuvant therapy	100 (95–100)	100 (95–100)	
DFS	Early-stage − Surgery alone	90 (79–100)	77 (62–92)	0.21
	Early-stage + Adjuvant therapy	100 (95–100)	100 (95–100)	
	Advanced-stage − Surgery alone	58 (42–74)	36 (20–52)	0.11
	Advanced-stage + Adjuvant therapy	85 (70–100)	80 (62–98)	
DSS	Advanced-stage − Surgery alone	72 (56–88)	60 (40–80)	0.59
	Advanced-stage + Adjuvant therapy	76 (58–94)	76 (58–94)	
OS	Advanced-stage − Surgery alone	58 (42–74)	48 (32–64)	0.69
	Advanced-stage + Adjuvant therapy	76 (58–94)	52 (34–70)	

Note: Abbreviations: DCD = distant control of disease, DFS = disease free survival, DSS = disease specific survival, LCD = local control of disease, KM = Kaplan-Meier; OS = overall survival, RCD = regional control of disease, CI = confidence interval.

### 3.2 Comparison Analysis between Early-Stage and Advanced-Stage Subgroups

Patients were stratified according to TNM 8th edition stage into early-stage (I and II stages, n = 28) and advanced-stage (III and IV stages, n = 27) groups (Table 1). The mean age at diagnosis did not differ significantly between cohorts (56.34 ± 12.37 vs. 57.15 ± 14.47 years, *p* = 0.55). No statistically significant differences were observed regarding sex distribution or exposure to common risk factors such as smoking and alcohol consumption. Presenting symptoms were broadly similar across groups. Tumor subsite distribution, however, differed: in the early-stage cohort, the supraglottis accounted for 54% of cases, followed by the glottis (33%) and subglottis (13%). In contrast, the advanced-stage group showed a predominance of supraglottic tumors (85%), with less frequent glottic (11%) and subglottic (4%) involvement. The supraglottic predominance in advanced disease was statistically significant (*p* = 0.02), suggesting an association between tumor site and more advanced presentation. High-grade histology (G3) was more frequent in advanced tumors (43% vs. 36% in early-stage, *p* = 0.04). Similarly, nodal metastases (N+) were significantly associated with advanced stage (56% vs. 0%, *p* < 0.001). Treatment also varied: total laryngectomy was more common in advanced cases (74% vs. 46% in early-stage, *p* = 0.32, while organ-preserving approaches (partial laryngectomy, cordectomy) were more often performed in early-stage disease. Adjuvant therapy was administered more frequently in advanced tumors, reflecting adverse pathological features.

### 3.3 The Role of Adjuvant Therapy in Early-Stage Laryngeal MEC

**Figure 2:**
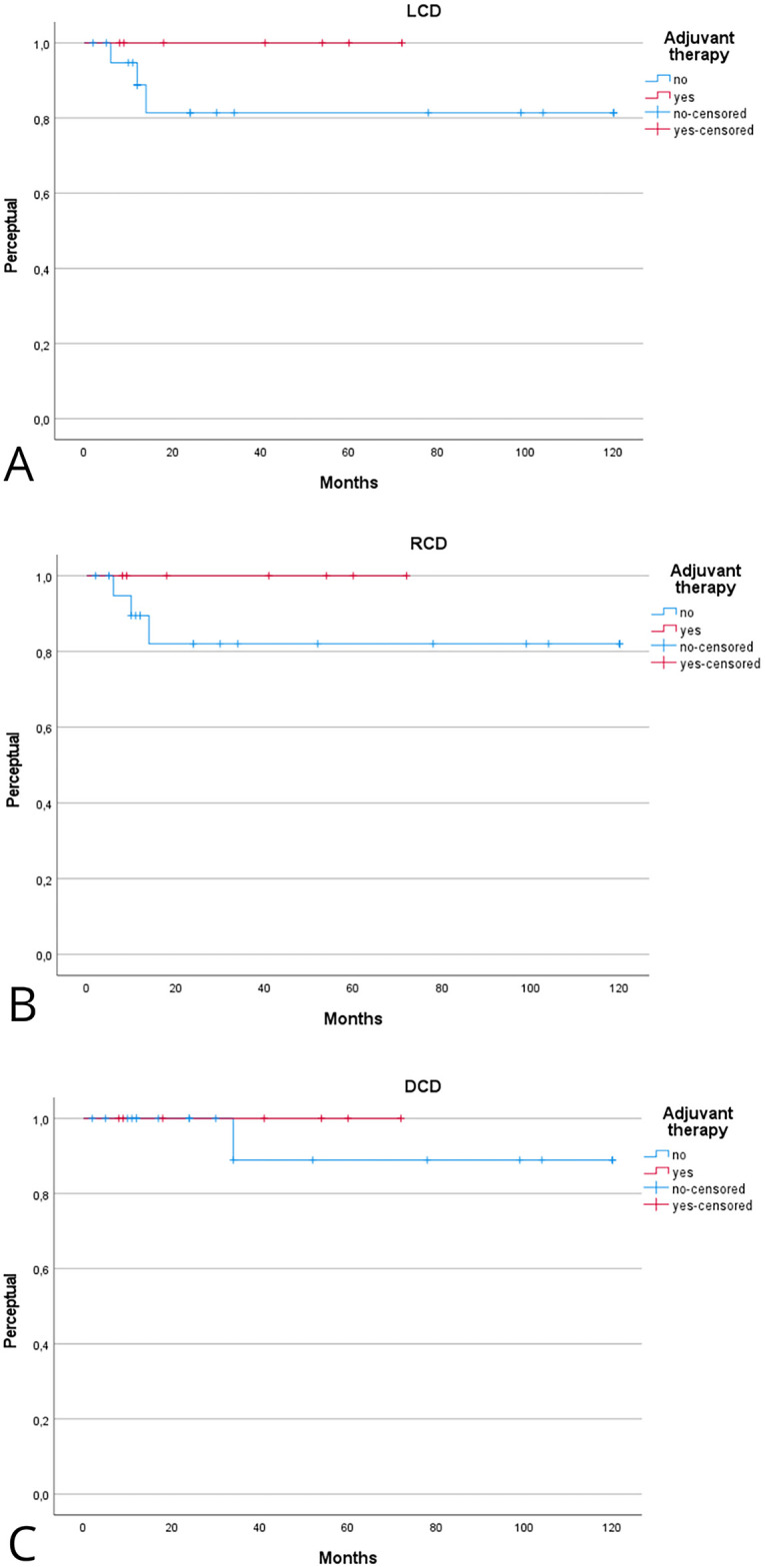
Kaplan–Meier analysis evaluating the effect of adjuvant therapy on local (**A**), regional (**B**), and distant (**C**) control of disease. Kaplan–Meier estimates are reported at 2 and 5 years with 95% confidence intervals (CIs), calculated using Greenwood’s variance and log–log transformation. Two-sided log-rank *p*-values are provided. Curves display the number of patients at risk below the *x*-axis, censoring marks along the survival lines, and time expressed in years. The median follow-up was 46 months (interquartile range, 30–72). A total of 11 local, 10 regional, and 5 distant recurrences were observed. Corresponding 2- and 5-year Kaplan–Meier estimates are detailed in Table 2. Abbreviations: LCD = local control of disease; RCD = regional control of disease; DCD = disease control of disease.

**Figure 3:**
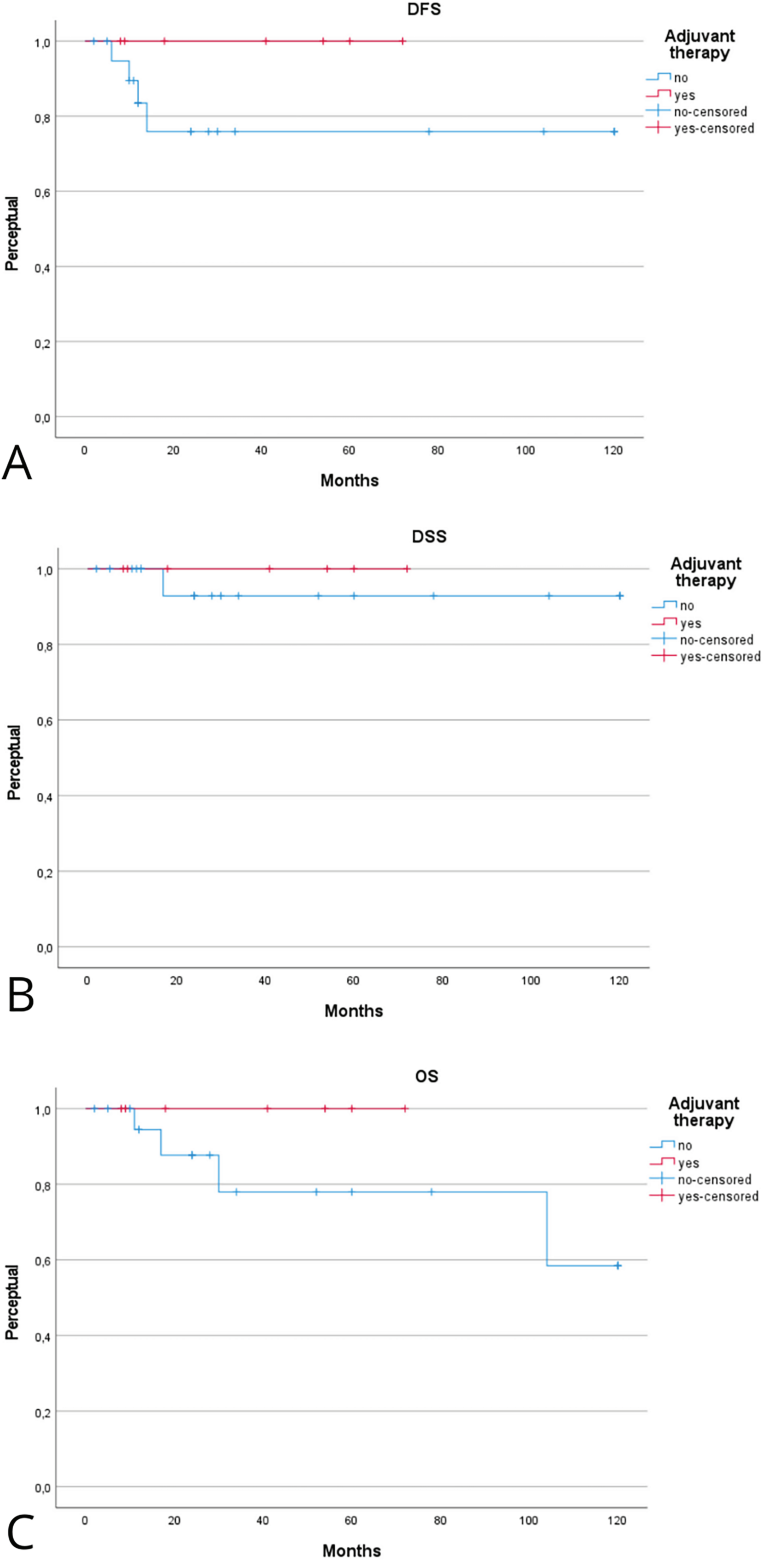
Kaplan–Meier analysis evaluating the effect of adjuvant therapy on disease-free (**A**), disease-specific (**B**), and overall (**C**) survival. Kaplan–Meier estimates are reported at 2 and 5 years with 95% CIs, calculated using Greenwood’s variance and log–log transformation. Two-sided log-rank *p*-values are provided. Curves display the number of patients at risk below the *x*-axis, censoring marks along the survival lines, and time expressed in years. The median follow-up was 46 months (interquartile range, 30–72). A total of 15 deaths occurred, including 9 disease-related. Corresponding 2- and 5-year Kaplan–Meier estimates are detailed in Table 2.

Patients receiving adjuvant therapy showed a consistent trend toward improved oncologic outcomes, although most differences did not reach statistical significance (Table 2). Fig. 2 shows the Kaplan–Meier analysis of disease control. At 5 years, Kaplan-Meier estimated LCD was higher in patients treated with adjuvant therapy compared with surgery alone (100% [95% CI, 95–100] vs. 85% [95% CI, 75–95]; log-rank *p* = 0.31). A similar trend was observed for regional and distant control, with the adjuvant group maintaining 100% control at 5 years, compared with approximately 80%–88% in patients treated with surgery alone. Fig. 3 illustrates survival outcomes. The 5-year Kaplan Meier DFS was 100% [95% CI, 95–100] in the adjuvant group and 77% [95% CI, 62–92] in the surgery-alone group (log-rank *p* = 0.21). No disease-related deaths occurred in patients who received adjuvant therapy, resulting in 5-year DSS and OS of 76% and 52% compared with 60% and 48%, respectively, in the surgery-only group. Although these differences did not reach statistical significance, they confirm that in early-stage, low-grade laryngeal MEC, complete surgical excision with negative margins provides excellent oncologic outcomes, and the additional benefit of adjuvant radiotherapy appears minimal.

### 3.4 The Role of Adjuvant Therapy in Advanced-Stage Laryngeal MEC

**Figure 4:**
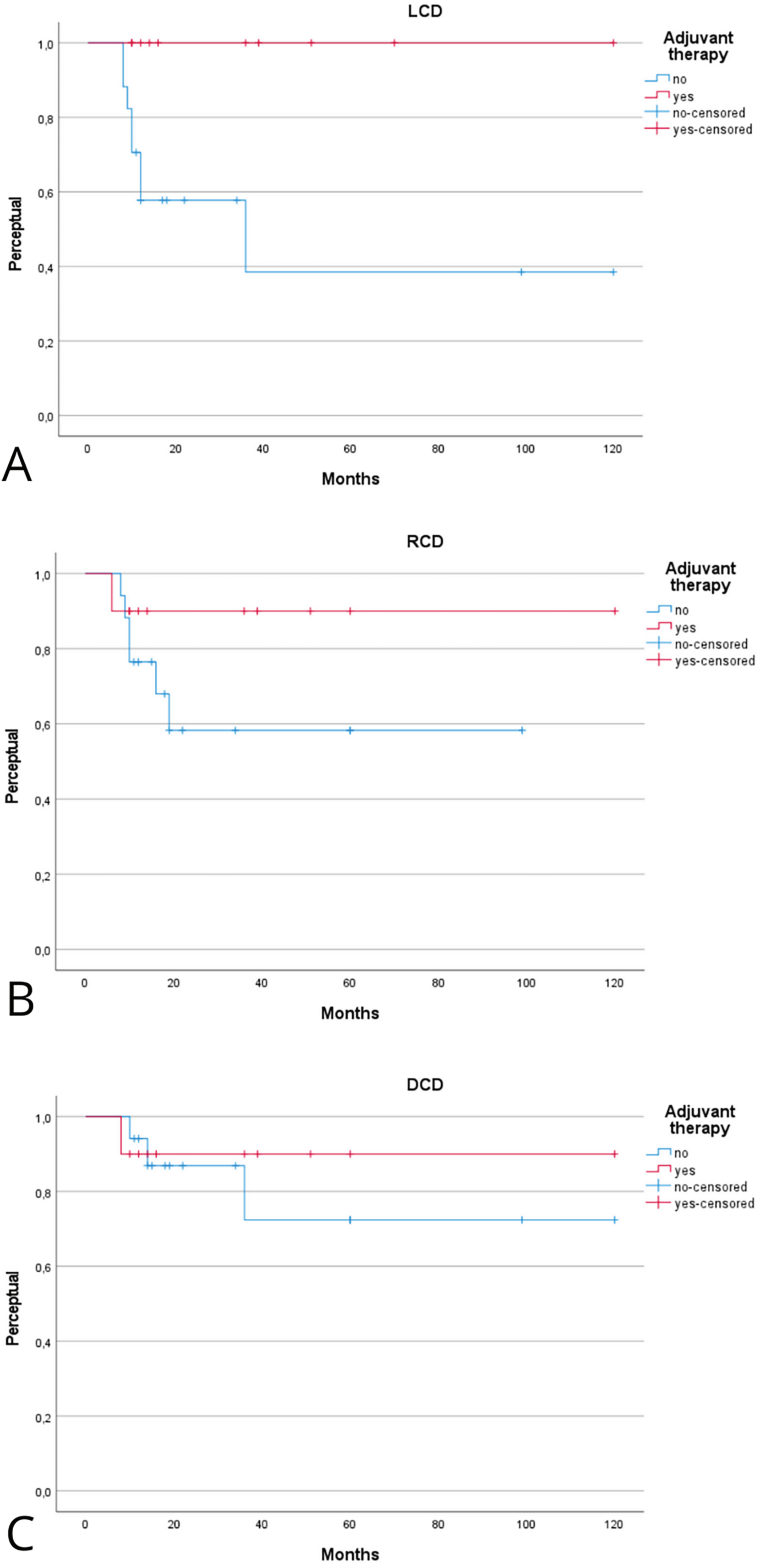
Kaplan–Meier analysis evaluating the effect of adjuvant therapy on local (**A**), regional (**B**), and distant (**C**) control of disease in advanced-stage laryngeal mucoepidermoid laryngeal cancer (MEC). Kaplan–Meier estimates are reported at 2 and 5 years with 95% CIs, calculated using Greenwood’s variance and log–log transformation. Two-sided log-rank *p*-values are provided. Curves include number-at-risk tables below the *x*-axis, censoring marks along the survival lines, and time expressed in years. The median follow-up was 46 months (interquartile range, 30–72). A total of 8 local, 7 regional, and 4 distant recurrences were observed. Corresponding 2- and 5-year Kaplan–Meier estimates are reported in Table 2.

**Figure 5:**
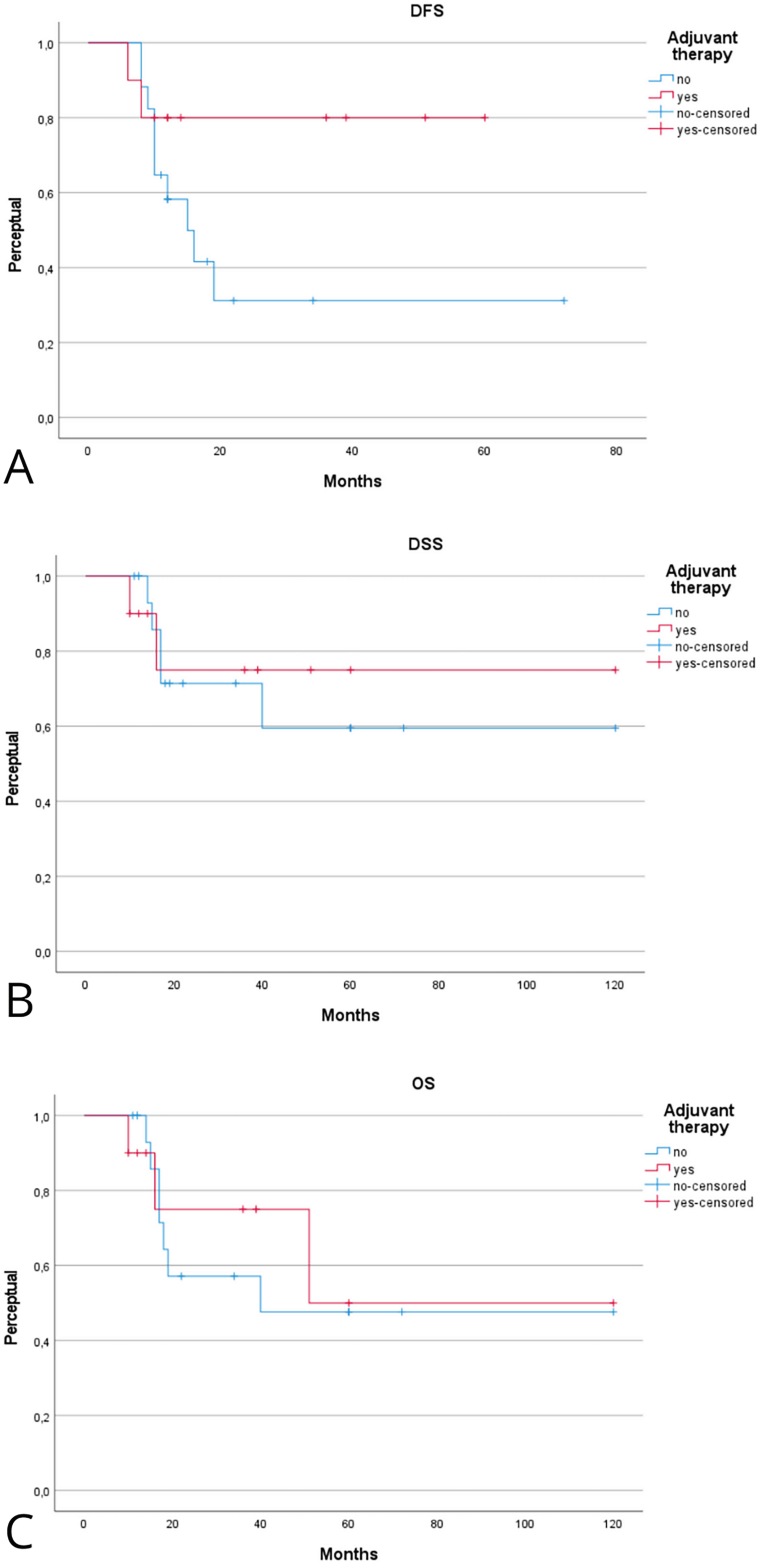
Kaplan–Meier analysis evaluating the effect of adjuvant therapy on disease-free (**A**), disease-specific (**B**), and overall (**C**) survival in advanced-stage laryngeal MEC. Kaplan–Meier estimates are reported at 2 and 5 years with 95% CIs, calculated using Greenwood’s variance and log–log transformation. Two-sided log-rank *p*-values are provided. Curves display censoring marks along the survival lines, numbers at risk below the time axis, and time expressed in years. The median follow-up was 46 months (interquartile range, 30–72). A total of 15 deaths occurred, including 9 disease-related. Corresponding 2- and 5-year Kaplan–Meier probabilities are reported in Table 2.

The prognostic role of adjuvant therapy in advanced-stage laryngeal MEC is summarized in Tables 2 and 3. Fig. 4 shows the Kaplan–Meier analysis of disease control. In advanced tumors, adjuvant therapy achieved markedly superior local control compared with surgery alone. The Kaplan-Meier 2- and 5-year LCD rates were 100% [95% CI, 95–100] with adjuvant therapy vs. 56% [95% CI, 40–72] and 38% [95% CI, 22–54] with surgery alone (log-rank *p* = 0.01). Regional control in early stage also favored the adjuvant group, with 5-year RCD of 100% [95% CI, 95–100] compared with 56% [95% CI, 36–76] in patients treated with surgery alone (log-rank *p* = 0.09). Differences in distant control in early stage were less pronounced, with 5-year DCD of 100% [95% CI, 95–100] vs. 78% [95% CI, 62–94] (log-rank *p* = 0.33). Fig. 5 illustrates survival outcomes. Five-year DFS was 80% [95% CI, 62–98] in the adjuvant group and 36% [95% CI, 20–52] in the surgery-only group (log-rank *p* = 0.11). Overall, these findings indicate that adjuvant therapy substantially improves local and regional control in advanced-stage or high-grade laryngeal MEC, while its impact on long-term survival remains uncertain.

**Table 3:** Crude (non–time-adjusted) recurrence and survival outcomes in patients with laryngeal mucoepidermoid laryngeal cancer.

Outcome	All Patients (n = 55)	Early Stage (T1–T2, n = 28)	Advanced Stage (T3–T4, n = 27)
Any recurrence, n (%)	15 (27%)	3 (10%)	12 (44%)
Loical recurrence, n (%)	11 (20%)	3 (10%)	8 (30%)
Regional recurrence, n (%)	10 (18%)	3 (10%)	7 (26%)
Distant metastasis, n (%)	5 (9%)	1 (4%)	4 (15%)
Disease-related deaths, n (%)	9 (16%)	2 (7%)	7 (26%)
All causes deaths	15 (27%)	5 (18%)	10 (37%)
Surgery alone	12 (30%)	3 (11%)	9 (43%)
Surgery + adjuvant therapy	3 (20%)	0 (0%)	3 (27%)

Note: Abbreviations: T = primary tumor site. Note: These data represent crude (non–time-adjusted) event proportions that do not account for censoring. They are provided for descriptive purposes and are not directly comparable with the Kaplan–Meier estimates reported. Abbreviations: DFS = disease free survival; DSS = disease specific survival; OS = overall survival.

## 4 Discussion

Laryngeal MEC is an extremely rare malignancy, representing <0.5% of all laryngeal cancers, with fewer than 150 cases described in the literature [2,3,8,24,32]. Given its rarity, most of the available data on treatment and prognosis derive from small case series and retrospective reviews, limiting the strength of evidence. Nevertheless, an emerging consensus highlights the pivotal role of adjuvant therapy, particularly in advanced-stage and high-grade tumors.

To our knowledge, within the scope of the current literature, this review represents the first systematic evaluation specifically comparing surgery alone vs. surgery plus adjuvant therapy in laryngeal MEC, with outcomes stratified by disease stage.

Adjuvant therapy was predominantly delivered as postoperative RT, while chemotherapy was used only in rare cases, usually in the presence of multiple adverse features such as positive margins or nodal metastases. Specifically, CHT-protocols were based on Platinum-based regimens, often combined with agents such as 5-fluorouracil or taxanes, though evidence for survival benefit remains anecdotal and limited to small case series [1,8].

For low-grade laryngeal MEC confined to early stages, surgical excision with negative margins is the treatment of choice, with limited evidence supporting the benefit of adjuvant RT. Spiro et al. [2] reported 13 patients, predominantly low-grade MEC, achieving >80% 5-year survival with surgery alone. Similarly, Ferlito et al. [3], in a clinicopathological study of 11 cases, found no local recurrences among patients treated conservatively when margins were negative. Karatayli-Ozgursoy et al. [4] also observed excellent LCD in early-stage low-grade laryngeal MEC with surgery alone, reinforcing that adjuvant treatment is unnecessary in this setting. These data suggest that in early disease, the addition of RT does not significantly improve DFS provided complete resection is achieved. The current data are consistent with these findings: in the early-stage subgroup, patients treated with surgery alone achieved a 5-year LCD of approximately 85%–88%, whereas those receiving adjuvant therapy demonstrated 100% local control, with no recurrences observed during follow-up. Similarly, 5-year DFS reached 77% after surgery alone compared with 100% when adjuvant therapy was administered. However, the differences did not reach statistical significance, suggesting that the potential benefit of RT in early-stage disease remains marginal, particularly in the context of complete surgical excision with clear margins.

The prognostic role of adjuvant therapy becomes more relevant in advanced or high-grade laryngeal MEC. Nielsen et al. [1], in a national Danish study, demonstrated that postoperative RT improved LCD in high-grade salivary-type carcinomas of the larynx, including MEC. Damiani et al. [8], in a series of 21 patients with MEC and adenosquamous carcinomas, reported that recurrence rates decreased from 47% after surgery alone to 19% when adjuvant RT was administered. The current analysis confirmed these observations: adjuvant treatment was associated with markedly higher LCD rates, reaching 100% at both 2 and 5 years, compared with 56% and 38%, respectively, in patients treated with surgery alone. The calculated odds ratio for recurrence confirmed the protective effect of adjuvant therapy (*p* = 0.01). Regional recurrence was also reduced by adjuvant therapy. In the current cohort, 5-year RCD was 90% compared with 56% after surgery alone, consistent with Chiari et al. [5], who reported regional recurrence rates of 20%–30% in the absence of postoperative treatment. Conversely, distant metastasis did not appear significantly affected by adjuvant therapy: we observed 5-year DCD rates of 90% with adjuvant therapy vs. 78% without it, a difference without statistical significance. Mahlstedt et al. [24] similarly reported distant metastasis rates of 10%–15% in high-grade MEC, irrespective of RT. The impact of adjuvant therapy on survival is less well defined, though a favorable trend is evident. In our cohort, 5-year DSS was 76% in patients receiving adjuvant treatment compared with 60% after surgery alone. Moreover, OS reached 52% in the adjuvant group vs. 48% in the surgery-only group. Although these differences did not reach statistical significance, they are clinically meaningful and in line with Damiani et al. [8], who described improved DFS but no clear OS advantage, likely due to small sample size and competing mortality factors. These findings suggest that adjuvant therapy provides meaningful improvement in local and regional control for advanced-stage or high-grade MEC, even though its impact on long-term survival remains uncertain.

Detailed information on RT protocols was rarely reported across the included studies. When available, adjuvant RT was typically delivered with conventional fractionation, using total doses ranging from 60 to 66 Gy in 2 Gy daily fractions to the primary tumor bed, with elective or therapeutic neck irradiation in cases with nodal involvement or high-grade histology. These parameters are consistent with standard postoperative regimens used for major salivary gland malignancies of the head and neck. Data regarding treatment volumes were limited, but most reports described inclusion of the laryngeal primary site and upper cervical lymphatics (levels II–III). Functional outcomes were seldom detailed. Among available cases, mild-to-moderate dysphonia and transient swallowing difficulties were the most frequently reported sequelae, mainly following total laryngectomy rather than as a direct effect of RT. No cases of radionecrosis or severe aspiration were described. These findings suggest that, when appropriately indicated and planned, postoperative RT for laryngeal MEC can be delivered safely, with acceptable functional outcomes and no clear evidence of long-term compromise in voice or swallowing function.

The present review provides an innovative contribution by being, to our knowledge, the first systematic analysis within our search scope to specifically compare surgery alone vs. surgery plus adjuvant therapy in laryngeal MEC, with outcomes stratified by stage, offering clinically oriented insights for decision-making.

## 5 Suggestions, Limitations, and Future Perspectives

The current evidence on laryngeal MEC is limited by the rarity of the disease and the consequent lack of prospective studies. Most available reports are retrospective case series including fewer than 20 patients, with variable follow-up and non-uniform definitions of histological grade and stage. Furthermore, historical misclassification of high-grade MEC as adenosquamous carcinoma or poorly differentiated SCC further complicates the interpretation of outcomes. In addition, the small number of patients included in this review (55 in total across 22 studies) represents the main limitation, together with the marked heterogeneity of reporting and the incomplete description of treatment details. These aspects further reduce the robustness of statistical comparisons and underline the descriptive rather than confirmatory nature of the present analysis. In positioning our work within the existing literature, it should be noted that prior systematic reviews—such as that by Chiari et al. (2024)—focused primarily on clinicopathologic features and prognostic factors rather than treatment comparison [5]. While the earlier study investigated prognostic factors such as stage and histopathology across all available cases, the present work specifically focuses on treatment outcomes and the role of adjuvant therapy, adopting revised inclusion/exclusion criteria and an updated literature search. This explains the partial overlap of source studies but also the different patient counts and treatment distributions. Future efforts should focus on multicenter collaborations with standardized diagnostic criteria and larger patient cohorts. In particular, the creation of prospective registries with harmonized staging and histological definitions would allow more reliable comparisons. It is important to note that the Kaplan–Meier curves presented in this review are reconstructed from aggregated case-series data rather than true individual-level survival times. Consequently, these analyses provide descriptive visualization rather than inferential survival statistics, and the resulting probabilities should be interpreted with caution. This limitation reflects the inherent constraints of the available evidence, which is dominated by small retrospective reports with heterogeneous follow-up. Furthermore, prospective registries employing uniform treatment protocols—including standardized indications for adjuvant RT and RT dose/fractionation schedules—would be instrumental in clarifying its true oncologic value. Integration of functional endpoints, such as swallowing and voice outcomes, should also be encouraged, as adjuvant RT may compromise quality of life even in the absence of clear survival benefit. Finally, novel therapeutic strategies, including precision RT techniques and immunotherapy-based approaches, warrant exploration for advanced-stage or recurrent MEC, where prognosis remains poor despite aggressive combined therapy.

## 6 Conclusions

Laryngeal MEC remains an exceptionally rare malignancy for which the current evidence base is limited to small, heterogeneous retrospective studies. While our findings suggest that postoperative radiotherapy may improve local and regional control in advanced-stage or high-grade disease, the certainty of this evidence is low, and no definitive survival advantage can be established. For early-stage, low-grade tumors, surgery with negative margins continues to appear sufficient, though this conclusion is likewise constrained by the low robustness of available data. Overall, treatment decisions should be individualized and made within a multidisciplinary context, recognizing that all therapeutic recommendations remain based on limited and low-certainty evidence. Larger collaborative studies and standardized reporting are needed to clarify the true impact of adjuvant therapy in this rare cancer.

## Supplementary Materials



## Data Availability

Data available on request from the authors.

## Supplementary Materials

The supplementary material is available online at https://www.techscience.com/doi/10.32604/or.2026.073086/s1.

## Abbreviations

Abb: Full Terms
AJCC: American Joint Committee on Cancer
CHT: Chemotherapy
MEC: Mucoepidermoid
RT: Radiotherapy
SCC: Squamous Cell Carcinoma
TOLM: Trans Oral Laser Microsurgery
TORS: Trans Oral Robotic Surgery
