# Supplementary material for: Surgery Alone Treatment vs. Surgery with Adjuvant Therapy for Laryngeal Mucoepidermoid Cancer: A Systematic Review
Source: Oncol Res. 2026 Mar 23;34(4):8. doi: 10.32604/or.2026.073086 (PMC13040313; doi:10.32604/or.2026.073086)
Supplement: Supplementary file 1 [file OncolRes-34-73086-s001.docx]

**Supplementary Table S1:** PRISMA 2020 Abstract - Check list.

| **Section and Topic** | **Item #** | **Checklist item** | **Reported (Yes/No)** |
| --- | --- | --- | --- |
| **TITLE** | | |  |
| Title | 1 | Identify the report as a systematic review. | Yes |
| **BACKGROUND** | | |  |
| Objectives | 2 | Provide an explicit statement of the main objective(s) or question(s) the review addresses. | Yes |
| **METHODS** | | |  |
| Eligibility criteria | 3 | Specify the inclusion and exclusion criteria for the review. | Yes |
| Information sources | 4 | Specify the information sources (e.g. databases, registers) used to identify studies and the date when each was last searched. | Yes |
| Risk of bias | 5 | Specify the methods used to assess risk of bias in the included studies. | Yes |
| Synthesis of results | 6 | Specify the methods used to present and synthesise results. | Yes |
| **RESULTS** | | |  |
| Included studies | 7 | Give the total number of included studies and participants and summarise relevant characteristics of studies. | Yes |
| Synthesis of results | 8 | Present results for main outcomes, preferably indicating the number of included studies and participants for each. If meta-analysis was done, report the summary estimate and confidence/credible interval. If comparing groups, indicate the direction of the effect (i.e. which group is favoured). | Yes |
| **DISCUSSION** | | |  |
| Limitations of evidence | 9 | Provide a brief summary of the limitations of the evidence included in the review (e.g. study risk of bias, inconsistency and imprecision). | Yes |
| Interpretation | 10 | Provide a general interpretation of the results and important implications. | Yes |
| **OTHER** | | |  |
| Funding | 11 | Specify the primary source of funding for the review. | Yes |
| Registration | 12 | Provide the register name and registration number. | Yes |

**Supplementary Table S2:** PRISMA 2020 - Check list.

| **Section and Topic** | **Item No** | **Checklist item** | **Reported on Page Number/Line Number** | **Reported on Section/Paragraph** |
| --- | --- | --- | --- | --- |
| **TITLE** | | |  |  |
| Title | 1 | Identify the report as a systematic review. | Page 1, line 1 | Title |
| **ABSTRACT** | | |  |  |
| Abstract | 2 | See the PRISMA 2020 for Abstracts checklist. | Page 2, lines 77,78 | Abstract |
| **INTRODUCTION** | | |  |  |
| Rationale | 3 | Describe the rationale for the review in the context of existing knowledge. | Page 2, lines 69-70 | 1. Introduction |
| Objectives | 4 | Provide an explicit statement of the objective(s) or question(s) the review addresses. | Page 2, lines 70-72 | 1. Introduction |
| **METHODS** | | |  |  |
| Eligibility criteria | 5 | Specify the inclusion and exclusion criteria for the review and how studies were grouped for the syntheses. | Page 3, lines 95-104 | 2.1. Search strategy and information sources |
| Information sources | 6 | Specify all databases, registers, websites, organisations, reference lists and other sources searched or consulted to identify studies. Specify the date when each source was last searched or consulted. | Page 3, lines 78-89 | 2.1. Search strategy and information sources  Supp table 3 |
| Search strategy | 7 | Present the full search strategies for all databases, registers and websites, including any filters and limits used. | Page 3, lines 78-89 | 2.1. Search strategy and information sources  Supp table 3 |
| Selection process | 8 | Specify the methods used to decide whether a study met the inclusion criteria of the review, including how many reviewers screened each record and each report retrieved, whether they worked independently, and if applicable, details of automation tools used in the process. | Pag 3, lines 105-109 | 2.2. Study selection and data extraction |
| Data collection process | 9 | Specify the methods used to collect data from reports, including how many reviewers collected data from each report, whether they worked independently, any processes for obtaining or confirming data from study investigators, and if applicable, details of automation tools used in the process. | Pag 3, lines 88-89 | Supp table 3 |
| Data items | 10a | List and define all outcomes for which data were sought. Specify whether all results that were compatible with each outcome domain in each study were sought (e.g. for all measures, time points, analyses), and if not, the methods used to decide which results to collect. | Pag 3, lines 100-102 | 2.2. Study selection and data extraction |
|  | 10b | List and define all other variables for which data were sought (e.g. participant and intervention characteristics, funding sources). Describe any assumptions made about any missing or unclear information. | Pag 3, lines 94-104 | 2.2. Study selection and data extraction |
| Study risk of bias assessment | 11 | Specify the methods used to assess risk of bias in the included studies, including details of the tool(s) used, how many reviewers assessed each study and whether they worked independently, and if applicable, details of automation tools used in the process. | Pag 4, lines 129-146 | 2.3. Quality assessment |
| Effect measures | 12 | Specify for each outcome the effect measure(s) (e.g. risk ratio, mean difference) used in the synthesis or presentation of results. | Pag 3, lines 100-102 | 2.2. Study selection and data extraction |
| Synthesis methods | 13a | Describe the processes used to decide which studies were eligible for each synthesis (e.g. tabulating the study intervention characteristics and comparing against the planned groups for each synthesis (item #5)). | Pag 3-4, lines 92-126 | 2.2. Study selection and data extraction |
|  | 13b | Describe any methods required to prepare the data for presentation or synthesis, such as handling of missing summary statistics, or data conversions. | Pag 5, lines 149-172 | 2.4 Data analysis |
|  | 13c | Describe any methods used to tabulate or visually display results of individual studies and syntheses. | Pag 5, lines 149-172 | 2.4 Data analysis |
|  | 13d | Describe any methods used to synthesize results and provide a rationale for the choice(s). If meta-analysis was performed, describe the model(s), method(s) to identify the presence and extent of statistical heterogeneity, and software package(s) used. | Pag 5, lines 149-172 | 2.4 Data analysis |
|  | 13e | Describe any methods used to explore possible causes of heterogeneity among study results (e.g. subgroup analysis, meta-regression). | Pag 5, lines 149-172 | 2.4 Data analysis |
|  | 13f | Describe any sensitivity analyses conducted to assess robustness of the synthesized results. | Pag 5, lines 149-172 | 2.4 Data analysis |
| Reporting bias assessment | 14 | Describe any methods used to assess risk of bias due to missing results in a synthesis (arising from reporting biases). | Pag 4, lines 129-146 | 2.3. Quality assessment |
| Certainty assessment | 15 | Describe any methods used to assess certainty (or confidence) in the body of evidence for an outcome. | Pag 4, lines 129-146 | 2.3. Quality assessment |
| **RESULTS** | | |  |  |
| Study selection | 16a | Describe the results of the search and selection process, from the number of records identified in the search to the number of studies included in the review, ideally using a flow diagram. | Pag 5, lines 175-184 | Figure 1 |
|  | 16b | Cite studies that might appear to meet the inclusion criteria, but which were excluded, and explain why they were excluded. | Pag 4, lines 135-139 | Supp table 4 |
| Study characteristics | 17 | Cite each included study and present its characteristics. | Pag 6, lines 190-191 | Supp table 4 |
| Risk of bias in studies | 18 | Present assessments of risk of bias for each included study. | Pag 4, lines 135-139 | Supp table 4 |
| Results of individual studies | 19 | For all outcomes, present, for each study: (a) summary statistics for each group (where appropriate) and (b) an effect estimate and its precision (e.g. confidence/credible interval), ideally using structured tables or plots. | Pag 6, lines 190-191 | Table 1 |
| Results of syntheses | 20a | For each synthesis, briefly summarise the characteristics and risk of bias among contributing studies. | Pag 14-15, lines 370-397 | 5. Suggestions, limitations, and future perspectives |
|  | 20b | Present results of all statistical syntheses conducted. If meta-analysis was done, present for each the summary estimate and its precision (e.g. confidence/credible interval) and measures of statistical heterogeneity. If comparing groups, describe the direction of the effect. | Pag 7-8, lines 218-246 | Table 2-3  Figure 2-5 |
|  | 20c | Present results of all investigations of possible causes of heterogeneity among study results. | Pag 14-15, lines 370-397 | 5. Suggestions, limitations, and future perspectives |
|  | 20d | Present results of all sensitivity analyses conducted to assess the robustness of the synthesized results. | Pag 7-8, lines 218-246 | Table 2-3  Figure 2-5 |
| Reporting biases | 21 | Present assessments of risk of bias due to missing results (arising from reporting biases) for each synthesis assessed. | Pag 14-15, lines 370-397 | 5. Suggestions, limitations, and future perspectives |
| Certainty of evidence | 22 | Present assessments of certainty (or confidence) in the body of evidence for each outcome assessed. | Pag 14-15, lines 370-397 | 5. Suggestions, limitations, and future perspectives |
| **DISCUSSION** | | |  |  |
| Discussion | 23a | Provide a general interpretation of the results in the context of other evidence. | Pag 13-14, lines 305-368 | 4. Discussion |
|  | 23b | Discuss any limitations of the evidence included in the review. | Pag 14-15, lines 370-397 | 4. Discussion |
|  | 23c | Discuss any limitations of the review processes used. | Pag 14-15, lines 370-397 | 4. Discussion |
|  | 23d | Discuss implications of the results for practice, policy, and future research. | Pag 14-15, lines 370-397 | 4. Discussion |
| **OTHER INFORMATION** | | |  |  |
| Registration and protocol | 24a | Provide registration information for the review, including register name and registration number, or state that the review was not registered. | Pag 3, lines 116-117 | 2.2. Study selection and data extraction |
|  | 24b | Indicate where the review protocol can be accessed, or state that a protocol was not prepared. | Pag 3, lines 116-117 | 2.2. Study selection and data extraction |
|  | 24c | Describe and explain any amendments to information provided at registration or in the protocol. | Pag 3, lines 116-117 | 2.2. Study selection and data extraction |
| Support | 25 | Describe sources of financial or non-financial support for the review, and the role of the funders or sponsors in the review. | Pag 15, line 409 | Funding statement  Ethical approval |
| Competing interests | 26 | Declare any competing interests of review authors. | Pag 15, line 417 | Conflict of interest |
| Availability of data, code and other materials | 27 | Report which of the following are publicly available and where they can be found: template data collection forms; data extracted from included studies; data used for all analyses; analytic code; any other materials used in the review. | Pag 15, line 415 | Availability of Data and Materials |

**Supplementary Table S3: Detailed Database Search Strategies and Retrieval Information.**

| Database | Search strategy (Boolean + controlled vocabulary) | Search dates | Gray literature inclusion | Filters applied | Records retrieved | Deduplication method |
| --- | --- | --- | --- | --- | --- | --- |
| PubMed | (“mucoepidermoid carcinoma” OR “mucoepidermoid tumor” OR “mucoepidermoid neoplasm” OR “salivary-type carcinoma” OR “sialogenic carcinoma”) AND (“larynx” OR “laryngeal”) **MeSH terms:** *Mucoepidermoid Carcinoma*; *Laryngeal Neoplasms* | July 5, 2025 | Reference lists of included studies were screened manually. No conference abstracts retrieved. | English only; humans | 274 | Exported to EndNote 21 → automatic removal of duplicates → manual check by two reviewers |
| Embase | ('mucoepidermoid carcinoma'/exp OR 'salivary gland-type carcinoma'/exp) AND ('larynx tumor'/exp OR 'laryngeal cancer'/exp) | July 5, 2025 | No additional gray literature available in Embase | English only; humans | 170 | Combined with PubMed and Scopus libraries → duplicate detection by EndNote 21 → manual verification |
| Scopus | TITLE-ABS-KEY(“mucoepidermoid carcinoma” OR “salivary-type carcinoma”) AND TITLE-ABS-KEY(“larynx” OR “laryngeal”) | July 6, 2025 | Books chapters and proceedings automatically indexed in Scopus were included | English only | 45 | Duplicates removed using EndNote 21 algorithm + manual review |
| Cochrane Library | (“mucoepidermoid carcinoma” AND “larynx”) | July 6, 2025 | No gray literature retrieved | No filters applied | 5 | Checked individually due to small number; compared with existing entries to avoid duplicates |
| Gray literature sources | Manual screening of reference lists of all included studies; no dedicated grey literature database used due to rarity of disease and expected absence of unpublished trials | July 2025 | Yes — reference list screening | — | 6 additional citations screened | Manual screening; duplicates compared with existing dataset |

**Supplementary Table S4: Quality Appraisal of Included Studies.**

| **Authors** | **Year** | **Selection** | **Ascertainment** | **Causality** | **Reporting** | **Risk of bias** |
| --- | --- | --- | --- | --- | --- | --- |
| **Ferlito [3]** | 1981 | 1 | 1 | 1 | 1 | **1** |
| **Damiani [8]** | 1981 | 1 | 1 | 1 | 0 | **2** |
| **Okinaka [11]** | 1984 | 1 | 1 | 1 | 1 | **1** |
| **Seo [26]** | 1980 | 1 | 1 | 1 | 1 | **1** |
| **Blinder [27]** | 1980 | 1 | 1 | 1 | 0 | **2** |
| **Gatta [25]** | 1980 | 1 | 1 | 1 | 1 | **1** |
| **Kaznelson [28]** | 1979 | 1 | 1 | 1 | 0 | **2** |
| **Tomita [29]** | 1977 | 1 | 1 | 1 | 0 | **2** |
| **Spiro [2]** | 1976 | 1 | 1 | 1 | 0 | **2** |
| **Skliris [30]** | 2023 | 1 | 1 | 1 | 0 | **2** |
| **Thomas [19]** | 1971 | 1 | 1 | 1 | 1 | **1** |
| **Mitchell [6]** | 1988 | 1 | 1 | 1 | 0 | **2** |
| **Alimoglu [13]** | 2011 | 1 | 1 | 1 | 1 | **1** |
| **Gomes [12]** | 1990 | 1 | 1 | 1 | 1 | **1** |
| **Calis [10]** | 2006 | 1 | 1 | 1 | 0 | **2** |
| **Nielsen [1]** | 2012 | 1 | 1 | 1 | 1 | **1** |
| **Prgomet [9]** | 1981 | 1 | 1 | 1 | 1 | **1** |
| **Tanaka [7]** | 2010 | 1 | 1 | 1 | 1 | **1** |
| **Zhang [31]** | 2014 | 1 | 1 | 1 | 0 | **2** |
| **Karatayli-Ozgursoy [4]** | 2016 | 1 | 1 | 1 | 0 | **2** |
| **Mahlsted [24]** | 2002 | 1 | 1 | 1 | 0 | **2** |
| **Yilmaz [32]** | 2013 | 1 | 1 | 1 | 0 | **2** |
| **Koike [18]** | 1979 | 1 | 1 | 0 | 0 | **3** |

The eight questions included in the evaluation tool are categorized into the following four domains: selection, ascertainment, causality and reporting. Within each domain the Authors evaluated whether the question related conditions were satisfied (1) or not (0). Final results differentiated case reports or series into 3 categories of risk of bias as follows: low risk (1) if all questions of the four domains were matched; intermediate risk (2) if questions of three out of the four domains were matched; high risk (3) if questions of less than three out of the four domains were matched.
